# Bone marrow aspirate concentrate/platelet-rich fibrin augmentation accelerates healing of aseptic upper limb nonunions

**DOI:** 10.1186/s10195-021-00582-y

**Published:** 2021-06-05

**Authors:** Alessandro Mazzotta, Cesare Stagni, Martina Rocchi, Nicola Rani, Nicolandrea Del Piccolo, Giuseppe Filardo, Dante Dallari

**Affiliations:** 1 Reconstructive Orthopaedic Surgery Innovative Techniques, Musculoskeletal Tissue Bank, IRCCS Istituto Ortopedico Rizzoli, via G.C. Pupilli, 1, 40136 Bologna, Italy; 2 Applied and Translational Research Center, IRCCS Istituto Ortopedico Rizzoli, via di Barbiano, 1/10, 40136 Bologna, Italy; 3 Via di Casaglia 28, 40135 Bologna, Italy

## Abstract

**Introduction:**

Nonunions remain a significant burden in orthopedics, often afflicting young males of working age. Positive findings have been published using bone marrow aspirate concentrate (BMAC) and platelet-rich fibrin (PRF) for the treatment augmentation of lower limb nonunions. The aim of this study was to investigate if the treatment augmentation with BMAC and PRF can also accelerate the healing of nonunions of the upper limb.

**Materials and methods:**

Sixty-eight patients (45 men, 23 women) affected by 75 nonunions of long bones of the upper limb were treated and divided into two groups. The first series was treated with standard surgery alone (group A); afterwards, the second series benefited from standard surgery with the addition of BMAC and PRF applied on lyophilized bone chips. Nonunions were classified radiographically according to the Weber–Cech method and prognostically using the Calori and Moghaddam scores. All patients were radiographically assessed at 1.5, 3, 6, 12, and 24 months of follow-up.

**Results:**

Baseline demographic characteristics did not present differences between groups. No differences were documented in terms of complications (two in group A and three in group B). Significant differences were instead documented in terms of healing time. The first healing signs were observed 1.5 months after surgery in 90.7% of patients in group B and 34.4% of group A (*p* < 0.0005). At 1.5, 3, 6, and 12 months, a higher radiographic score was found for group B (all *p* < 0.0005), while no difference was found at final follow-up of 24 months (90.6% of group A and 97.7% of group B achieved radiological healing). Faster healing with BMAC/PRF augmentation was confirmed for all bones, as well as for the subgroup of patients affected by atrophic nonunions (*p* = 0.001).

**Conclusion:**

This study showed the benefits of restoring both mechanical and biological aspects when addressing nonunions of the long bones of the upper limb. In particular, the association of BMAC and PRF to lyophilized bone chips was safe and able to accelerate healing time. These good results were confirmed for humerus, radius, and ulna sites, as well as for challenging atrophic nonunions of the upper limb.

## Introduction

Nonunions remain a significant burden in orthopedics. Despite advancement in the therapeutic procedures to address fractures, nonunion incidence has been stable over the years, accounting for 2–30% of overall fractures [1]. This has important clinical, social, and economic implications. An epidemiological study carried out in 2013 in Scotland [2] reported that the direct cost of treating long-bone nonunions is between £7000 and £79,000 per patient. Indirect costs can be even higher, accounting for up to 83–93% of the total cost of fracture treatment [3]. Furthermore, they often afflict young males of working age, in particular forearm nonunions [4]. Consequently, the overall health and social impacts are high owing to the long-lasting therapies and absence from work [5], and current research efforts are aimed at improving the healing potential and recovery time.

The main factors necessary to obtain bone healing are proper mechanical stabilization and biology, which may present a different relevance depending on the type of nonunion [6, 7]. In fact, nonunions are defined either as hypertrophic, hypervascularized, and vital and mainly caused by inadequate immobilization, or as atrophic, hypovascularized, and not vital with the absence of callus. The latter is therefore the most difficult to manage, and replacement of the fixation devices may not be sufficient [8]. Nonunion treatment usually requires resection of the nonunion area [9], even though bone shortening can have important biomechanical implications in particular at the forearm, where the distortion of the radio-ulnar ring can irreparably compromise pronosupination [10]. To address the issue of bone loss, the use of autologous grafts such as iliac crest or vascularized fibula [11–14] has been proposed, as they combine biological and mechanical properties [15]. However, these solutions encounter significant problems due to scarce graft availability and donor site comorbidity [16], hence the idea to use biological adjuvants with different properties in terms of osteogenesis, osteoinduction, and osteoconduction on a properly prepared well-vascularized surgical bed acting as a biological chamber [17, 18]. Positive findings in bone regeneration have been previously published on the application of bone marrow aspirate concentrate (BMAC) [19] and platelet-rich fibrin (PRF) [20]. Faster healing has been shown using BMAC and PRF in combination with homologous bone chips for the treatment of lower limb nonunions [21]. However, the literature lacks data on the efficacy of this strategy for nonunions of the upper limb.

Thus, the aim of this study was to investigate if the treatment augmentation with BMAC and PRF can accelerate the healing of nonunions of the upper limb.

## Materials and methods

This study was approved by the local ethics committee (protocol 0013446 (n. 684/2019/Oss/IOR)). All consecutive cases of aseptic nonunions of long bones of the upper limb treated surgically with internal fixation devices in the division of the Rizzoli Orthopaedic Institute in the period September 2000 to October 2017 were documented. Patients were included who had a diagnosis of posttraumatic nonunion defined as the clinical persistence of pain and/or preternatural motility and radiographic persistence of the fracture line with the absence of progression of reparative phenomena for at least 3 months. Patients under 18 years of age, or with body mass index (BMI) > 35 were excluded, as well as cases of pathological, septic nonunion, and those treated with external fixation devices.

According to these criteria, 75 consecutive patients (51 men, 24 women) were selected for a total of 83 cases of nonunions. Out of these patients, 7 patients (for a total of 8 nonunions) were lost to follow-up, for a total of 68 patients and 75 nonunions evaluated for the purpose of this study (34 nonunions of the humerus, 16 of the radius, and 25 of the ulna). The type of surgery depended on the year of treatment, since before 2005 the methods for the collection and processing of biological adjuvants were not available in our Institute. Accordingly, patients were divided into two groups (A and B) based on the treatment modality. Both groups were treated with standard surgery based on internal fixation devices such as plates and screws and bone grafts such as cortical homoplastic stick and/or lyophilized bone chips. Group B also benefited from augmentation with biological adjuvants BMAC and PRF.

### Treatment and evaluation

**Fig. 1:**
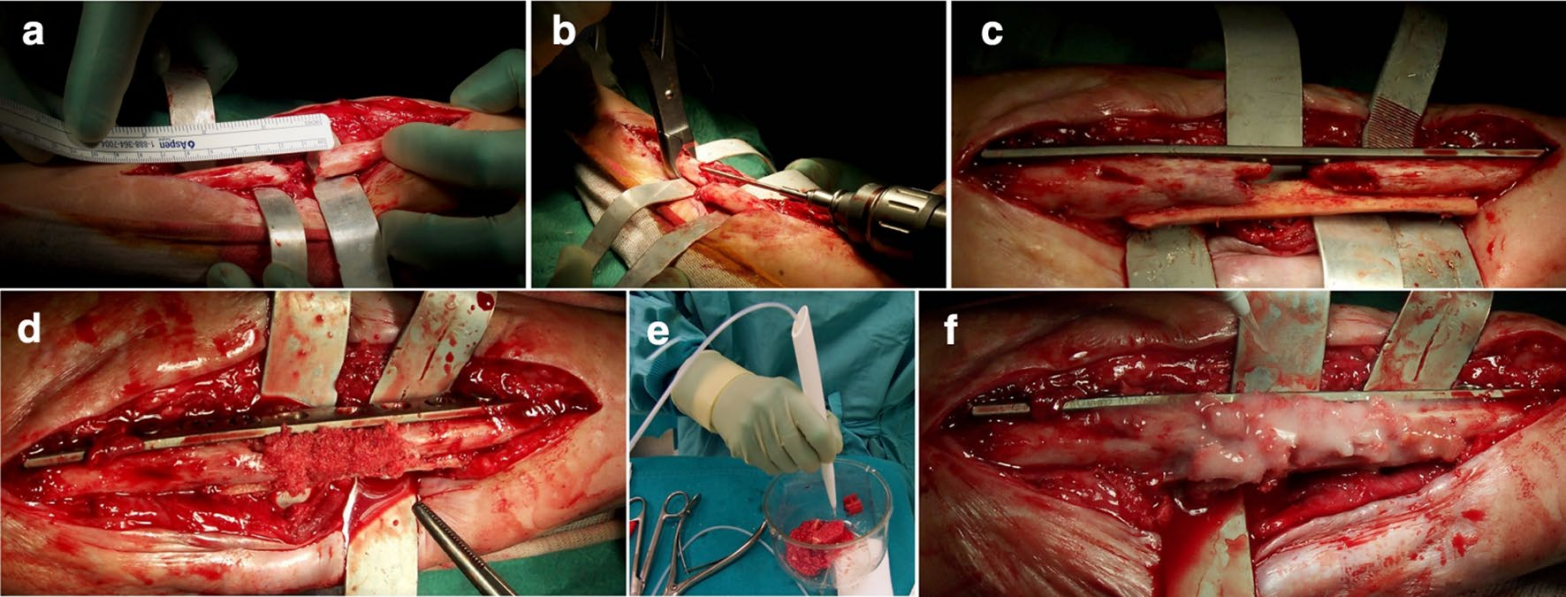
**a** Intraoperative images show an ulna atrophic nonunion with bone loss and consequent gap; **b** both groups of patients were treated with the standard surgery procedure consisting of removing nonviable bone tissue and reopening the medullary canal; **c** fractures were stabilized with plates and screws according to the AO principles and a cortical homoplastic stick to increase stability; **d** lyophilized bone chips were used to fill the gap; **e** preparation and **f** application of biological adjuvants (PRF and BMAC) in all patients of group B

Patients underwent mixed anesthesia, combining brachial plexus block with general anesthesia. Antibiotic prophylaxis consisted of intravenous administration of cefazoline 2 g during the induction phase. The patient position and surgical approach depended on the location of the nonunions. The surgical approach was chosen with the aim of respecting the soft tissues or the flaps of previous reconstructive surgeries. Proceeding by planes and isolating the vascular-nerve structures, the fracture was then exposed. Previous fixation devices and nonviable bone tissues were removed until biological active and bleeding bone was obtained. The medullary canals were reopened. The fracture segments were then modeled so that it was possible to achieve an anatomical reduction where possible, in particular for the forearm. In any case, being diaphyseal and metadiaphyseal fractures, the primary objective was to obtain the restoration of axis, length, and rotation. Occasionally the fractures were temporarily stabilized with K-wires or forceps. Fractures were definitely stabilized with plates and screws according to the AO principles with at least six cortical grip points for each part. In 69 cases (in 29 patients of group A and in 40 patients of group B), a cortical homoplastic stick was used to increase the stability of the fracture. In all cases in each group, freeze-dried homologous bone chips were used to fill the fracture gap. In all cases of group B, freeze-dried homologous bone chips acted as a vehicle for biological adjuvants (BMAC and PRF) (Fig. 1). At the end of surgery, careful hemostasis was performed to avoid the use of drainage. If necessary, a nonsuction drain was positioned, to avoid the loss of biological adjuvants.

The lyophilized bone chips were retrieved from bone of deceased donors and prepared according to a technique published elsewhere [22]. All organic material, such as blood, bone cells, and adipose tissue, was removed by subsequent washes of distilled water, chloroform, methanol, and oxygenated water. After a final soak, the bone was frozen at −80 °C overnight. BMAC was obtained through the BMAC Harvest System (Terumo, Munich, Germany), which allowed the separation of the buffy coat resulting from a withdrawal of 60 mL of bone marrow, taken with heparin (500 U in 10 mL of saline solution) from the ipsilateral iliac crest during the first phase of surgery and subsequently centrifuged at 3200 rpm for 10 min. PRF was prepared through the Vivostat PRF1 automatic system (Steripolar AB, Uppsala, Sweden) to obtain a high concentration of platelets and released growth factors. It was prepared without addition of thrombin to get a more elastic three-dimensional fibrin framework for favoring cell migration and the retention of circulating growth factors, to maintain its activity for a longer period and stimulate tissue regeneration more physiologically [23]. PRF was prepared 1 or 2 days before surgery: an autologous venous blood sample of 120 mL was withdrawn and centrifuged without anticoagulants at 3000 rpm for 10 min. PRF was thawed before surgery and naturally activated through application on the lyophilized bone chips. The combination of BMAC and PRF was applied on the lyophilized bone chips, previously rehydrated in saline solution. Thus, the biological adjuvant mixture was inserted into the nonunion site after stabilization, and the wound was closed.

The radiographic and prognostic characteristics of the nonunions within the two groups were analyzed through the classification of Weber and Cech and through the prognostic evaluation scores suggested by Calori [24, 25] and Moghaddam [26]. The latter take into account the prognostic characteristics of the fracture, which can be assessed by radiographic observation (bone quality, anatomical site, previous fixation devices, signs of mobilization, bone lost, Weber–Cech classification); the condition of the soft tissues, traceable by the physical examination described during hospitalization; and the characteristics of the patient [ASA, comorbidities as diabetes, inflammatory indices, drugs taken as nonsteroidal anti-inflammatory drugs (NSAIDs) and cortisones, smoking], described in the anamnesis, in the anesthesia and therapeutic record. All patients were clinically and radiographically checked at 1.5, 3, 6, 12, and 24 months after surgery or until consolidation. Clinically, the progressive disappearance of pain was considered. For radiographical evaluation, a previously published method was used [21]: standard anterior–posterior (AP)and lateral–lateral (LL) radiographs were blind-examined by two surgeons (D.D. and A.M.) through a score from 0 to 4, according to the parameters reported in Table 1.

**Table 1:** Radiological scoring system

Score	Definition
*0*	Visible non-union edge, no callus
*1*	Visible non-union edge, callus
*2*	Callus, visible non-union edge, early bone bridge and/or initial bone graft integration
*3*	Visible non-union edge, bone bridge and/or completed bone graft integration
*4*	Healed: no more visible non-union edge

## Statistical analysis

All continuous data are expressed in terms of mean ± SD, and categorical variables are expressed as proportions or percentages. The Shapiro–Wilk test was performed to test normality of continuous variables. The Levene’s test was used to assess the homoscedasticity of the data. Repeated-measures analysis of variance (ANOVA) was performed to compare the scores at different follow-up times. One-way ANOVA was performed to assess between-group differences of continuous and normally distributed and homoscedastic data; the Mann–Whitney test was used otherwise. Pearson *χ*^2^ exact test was performed to investigate relationships between grouping variables.

For all tests, *p* < 0.05 was considered significant. All statistical analysis was performed using SPSS v.19.0 (IBM Corp., Armonk, NY, USA).

## Results

Baseline demographic characteristics of the evaluated patients are reported in detail in Table 2. Group A and B did not significantly differ in the evaluated parameters in terms of age, sex, BMI, and radiographic characteristics according to Weber and Cech or prognostic features according to Calori and Moghaddam. No differences were detected in terms of complications: after surgery, two patients treated for humerus nonunion in group B developed a transient radial nerve deficit that healed by follow-up, and two patients in group A and 1 patient in group B (all treated for ulna nonunions) developed wound dehiscence, treated and healed through wound irrigation and oral antibiotic therapy.

**Table 2:** Demographic data

Characteristics	Group A (standard surgery)	Group B (standard surgery + PRF + BMAC)	*p* value
Patients	30	38	
Sex	19 male	26 male	0.456
	11 female	12 female	
Age (years)	42.4 ± 14.6	45.9 ± 12.4	0.226
BMI	26.4 ± 4.9	26.0 ± 3.5	0.881
Nonunions	32	43	
Site	18 humerus	17 humerus	0.363
	6 radius	11 radius	
	8 ulna	15 ulna	
Atrophic	28	40	0.451
Hypertrophic	4	3	
Calori	32.5 ± 10.0	33.4 ± 8.0	0.475
Moghaddam	16.7 ± 7.2	16.3 ± 8.8	0.716

**Fig. 2:**
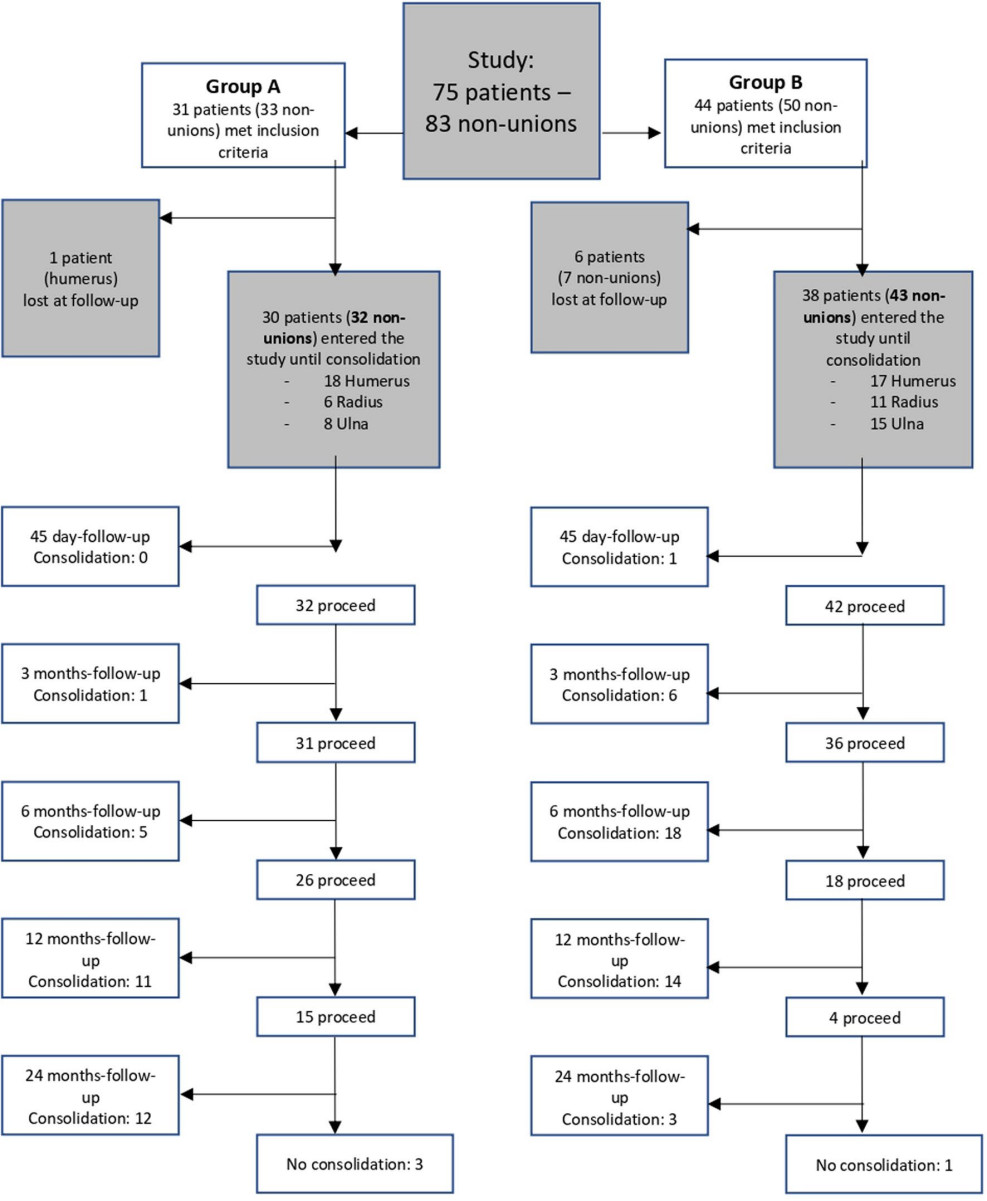
Flowchart summarizing participants, withdrawals, timing, and outcomes. Standard treatment procedure (group A); standard treatment procedure augmented with BMAC/PRF (group B)

Significant differences were instead documented in terms of healing time. A flow chart summarizing participants, withdrawals, timing, and outcomes is shown in Fig. 2.

**Fig. 3:**
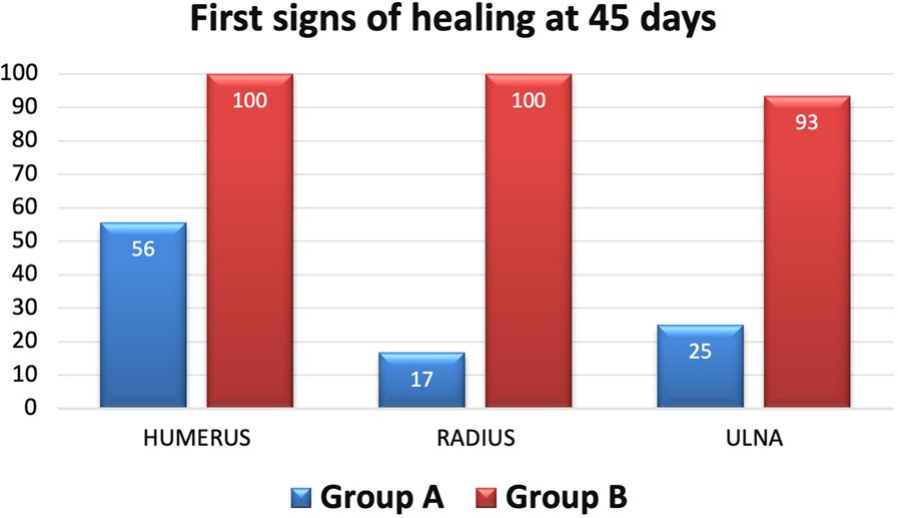
First signs of healing at 45 days after surgery comparing different anatomical sites in groups A and B

**Fig. 4:**
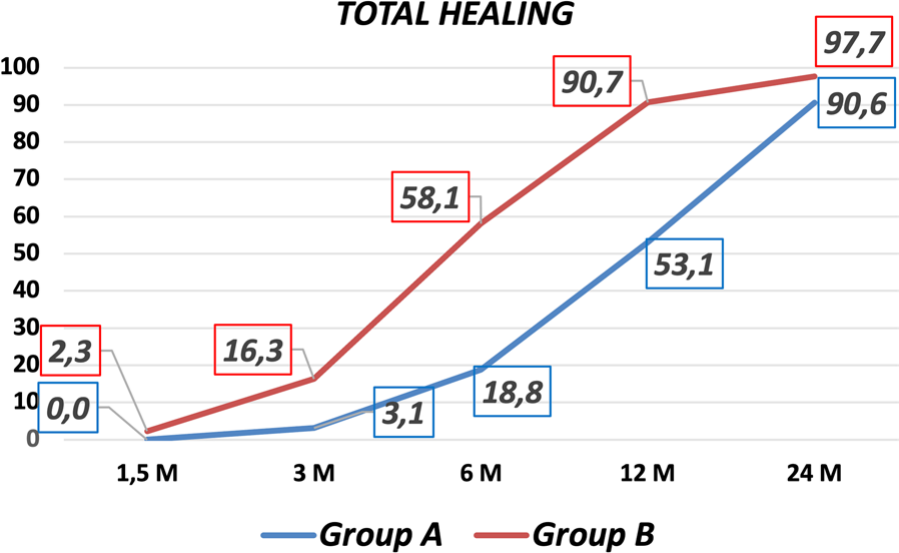
Percentages of radiological healing in group A (treated with the standard procedure) and group B (augmented with PRF/BMAC) at 1.5, 3, 6, 12, and 24 months of follow-up

The first signs of healing, equal to at least grade 1 according to the radiological classification, were observed 1.5 months after surgery in 90.7% of patients in group B and 34.4% of group A (*p* < 0.0005), as reported in detail in Fig. 3. At 1.5 months, a higher radiographic score was found for group B (*p* < 0.0005), although only one patient fully healed in group B versus none in group A. At 3 months, a higher radiographic score was found for group B (*p* < 0.0005), with 16.3% fully healed patients versus 3.1% in group A. At 6 months, a higher radiographic score was found for group B (*p* < 0.0005), with 58.1% fully healed patients versus 18.8% in group A. At 12 months, a higher radiographic score was found for group B (*p* = 0.004), with 90.7% fully healed patients versus 53.1% in group A. At final follow-up of 24 months, 97.7% subjects of group B (n.s) and 90.6% subjects of group A achieved radiological healing (Fig. 4). Kaplan–Meier analysis confirmed overall faster healing for group B (*p* < 0.0005). All patients who reached radiographic healing also presented no pain at the fracture site at final evaluation. The only not consolidated case in group B was an atrophic radius nonunion (with a score of 46 for Calori and 16 for Moghaddam). In group A, three cases did not consolidate: one of them was an atrophic ulna with bone loss > 2 cm due to the treatment of a previous infection (40 for Calori and 12 for Moghaddam); the second one was a patient with previous plastic surgery due to fracture exposition with neurological deficit (Calori 38; Moghaddam 18) that healed 1 year after the end of the study through further treatment with platelet-rich plasma (PRP) injections; the last patient was a woman with an atrophic humerus nonunion (Calori 40; Moghaddam 8) that developed graft and fixation device failure and was later reoperated and finally healed with biological adjuvants.

A further analysis was performed to independently consider humerus, radius, and ulna: the augmentation with BMAC/PRF improved the healing at every follow-up in all treatment locations (humerus, *p* = 0.023; radius, *p* = 0.057; ulna, *p* = 0.015). Moreover, hypertrophic nonunions were excluded to separately analyze atrophic nonunions, confirming also for this type of nonunion that patients treated with adjuvants had a significant acceleration of the consolidation in comparison with subjects treated with the standard procedure (*p* = 0.001). Finally, according to the analysis performed to investigate the role of patient and disease factors, healing time was not influenced by age, sex, BMI, smoke, site, trauma energy, fracture exposure, atrophic versus hypertrophic, previous treatments, or Calori and Moghaddam severity scores in this series.

## Discussion

The main finding of this study is that biological augmentation with BMAC/PRF was able to accelerate the healing of upper-limb nonunions.

Despite the advancement in fracture management, both in the diagnostic and therapeutic field, nonunions remain a significant burden in orthopedics, with important clinical, social, and economic implications. A suggestion to address this challenge was proposed in 2007 by Giannoudis et al. [6] through the diamond concept. This concept encompasses all the aspects of “polytherapy” [27, 28] that have been gaining ground in the literature in recent years, combining biomechanical and biological factors to promote fracture healing. In this light, after removal of nonviable tissue to create a biological chamber [17] with a good vascular bed to guarantee supply of oxygen and nutrients, as well as to ensure fracture stability with proper fixation devices, the therapeutic objective of biological adjuvants is to reactivate the “bioreactor” of bone healing [29]. The role of biological adjuvants, such as cells with osteogenic capacities, growth factors with osteoinductive properties, and scaffolds with three-dimensional structures to guide bone regeneration, has already been shown in the literature. BMAC [19], PRP [30, 31], or growth factors such as rhBMP [32] have been used alone or in combination with bone graft, with successful results in bone healing. Moreover, the literature suggests their combination might be beneficial, with BMAC and PRF together amplifying bone differentiation. For example, Perut et al. [33] studied the properties of PRP, showing that the presence of leukocytes led to considerably greater differentiation of BMAC in bone tissue. Moreover, PRF offers a tridimensional structure with proinflammatory cytokines able to induce leukocyte degranulation and, consequently, bone differentiation [23].

Based on these principles, encouraging results have been observed in the treatment of nonunions in the lower limb [34–36]. In particular, based on the promising findings, Dallari et al. [21] in a recent study on the treatment of lower limb nonunions used an association of biological adjuvants consisting of BMAC, PRF, and homologous bone chips. The augmentation showed a significantly faster healing time, and an improved consolidation grade was demonstrated also in the more challenging atrophic nonunions. This approach led to positive results also when considering complex upper limb cases, where a greater number of nonunions are documented, with even more atrophic nonunions [2]. Calori et al. [27] treated 52 forearm nonunions, dividing them into two treatment groups. The first one was managed according to the principles of “monotherapy” and the second using the “polytherapy” obtained with the association of rhBMP-7, autologous MSCs, and a scaffold. The healing rate at 12 months was 64% and 89%, respectively. Miska et al. [37] treated 50 patients with nonunions of the humeral shaft. The type of treatment was chosen on the basis of the prognostic factors of the individual cases, requiring in only six patients treatment with all the factors of the diamond concept (revision of the synthesis, BMAC, rhBMP-7, scaffold). Overall, healing was 80%; however, no comparison was made between patients treated with polytherapy and others, impairing the possibility to identify the specific contribution of biological augmentation. Being a combination of factors, the role of each product remains often difficult to be properly quantified. Moreover, similarly to the lower limb, different studies adopted a combination of different biological augmentation strategies [27, 37].

The ideal combination of biological factors is still debated. Calori et al. [38] compared two groups of patients affected by long-bone nonunions, one treated with rhBMP-7 and the other one with PRP, and obtained 87% versus 68% healing rates. However, PRP prepared with anticoagulants requires activation with thrombin, which causes a massive and short-lasting release of growth factors [39]. On the contrary, PRF is obtained with centrifugation without addition of anticoagulants [40] and presents a much slower and more natural activation, so that the release of growth factors and cytokines, such as PDGF, TGFB-1, and IGF-1, accompanies bone healing in a more physiological way. In addition, PRF has a more elastic three-dimensional structure compared with PRP, which favors cell migration and retention of circulating growth factors [23]. In vitro and in vivo studies have shown how these bioactive molecules are able to stimulate the differentiation of multipotent cells in bone tissue [41, 42]. The relevance of platelet concentrates as biological adjuvants is also suggested by some studies showing good results with PRP applied for enhancing bone healing [43], and also in this study it is interesting to observe the outcome of the only patient who did not heal in group B, who healed after injective treatment with three injections of PRP.

In this study, PRF was used as a blood derivative together with BMAC, as combined biological augmentation to foster the diamond concept and favor tissue healing. The upper limb has important biomechanical differences compared with the lower limb, being mainly used in distraction and rotation. Therefore, fixation devices such as plate and screws were preferred because intramedullary nailing might not offer the same guarantees found for the lower limb in terms of fracture compression and stability. In fact, although in acute fractures the use of intramedullary or extramedullary fixation devices can give similar results, McCormack et al. [44] showed that, for the revision procedures of nonunions, the use of compression plates should be the gold standard treatment. Following this approach, together with BMAC and PRF, the restoration of the mechanical and biological aspects led in this study to faster healing of group B (97.7%) compared with group A (93.3%). Of interest, one patient for whom the surgical treatment without biological adjuvants failed recovered following a second surgical treatment with the addition of BMAC and PRF. The good results offered by biological augmentation were confirmed for all anatomical sites and by the separate analysis of the results excluding hypertrophic nonunions, thus confirming that these adjuvants can restore the deficient biological factors also in challenging atrophic nonunions.

This study has some limitations, in particular because of its retrospective nature. As a retrospective study, it was not possible to use a clinical evaluation score to estimate progressive functional recovery of the patients. However, the study focused on radiographic healing, which was available for the treated patients and could be evaluated blindly. With regard to radiographical assessment, it should be noted that in the literature there is no “gold standard” for assessing the healing of nonunions. Second-level investigations have been proposed, such as computed tomography(CT) [45], scintigraphy [46], positron emission tomography–computed tomography (PET-CT) [47], but they are not easily available, they increase costs, and they are more invasive owing to the use of more X-rays or radio isotopes. X-rays are the fastest and most widely used, although they might present interpretative difficulties. Still, two authors of this study separately analyzed and graded the outcome, confirming an excellent rating agreement. Finally, the number of evaluated patients did not allow further subanalysis to investigate the influence of specific patient- and fracture-related factors in terms of healing time and response to the treatment adjuvants.

Despite the aforementioned limitations, this study is of clinical relevance for an important patient category. In fact, while bone defects over 6 cm seem to be better managed by a vascularized bone graft [48], the most common lesions within 6 cm are currently addressed by applying nonvascularized bone grafts. This study does not challenge this treatment cut-off. On the other hand, it showed that the use of biological adjuvants can accelerate the healing processes of lesions up to 6 cm in the upper limb. A recent literature review [49] highlighted that many articles underlined the potential of growth factors and bone marrow concentrates in the treatment of nonunions, as well as their synergistic effect, showing promising results for bone regeneration. While future high-level studies with larger case series are still needed, the results of this work add to this growing body of literature, confirming the encouraging results. This study is one of the largest available comparative studies in the field and was able to document a significantly faster healing time with BMAC and PRF, supporting the use of this biological augmentation strategy for the treatment of upper-limb nonunions.

## Conclusion

This study showed the benefits of restoring both mechanical and biological aspects when addressing nonunions of the long bones of the upper limb. In particular, the association of BMAC and PRF to lyophilized bone chips was safe and was able to accelerate the healing time. These good results were confirmed for humerus, radius, and ulna sites, as well as for challenging atrophic nonunions of the upper limb.

## Data Availability

The datasets generated and/or analyzed during the current study are not publicly available and are currently archived according to current regulations (with full personal details of all participating subjects) at the Rizzoli Orthopedic Institute, Bologna, Italy. All the datasets are available (after conversion to anonymous form) from the corresponding author on reasonable request.
